# C1222C Deletion in Exon 8 of *ABL1* Is Involved in Carcinogenesis and Cell Cycle Control of Colorectal Cancer Through IRS1/PI3K/Akt Pathway

**DOI:** 10.3389/fonc.2020.01385

**Published:** 2020-08-11

**Authors:** Yi Liu, Jian Cao, Ya-Ning Zhu, Yu Ma, Ghulam Murtaza, Yu Li, Jian-Hua Wang, Yan-Song Pu

**Affiliations:** ^1^Department of Oncology, Shaanxi Provincial People's Hospital, Xi'an, China; ^2^Department of Pharmacy, Xijing Hospital, Air Force Military Medical University, Xi'an, China; ^3^Department of Pharmacy, Shaanxi Provincial People's Hospital, Xi'an, China; ^4^Department of Pathology, Shaanxi Provincial People's Hospital, Xi'an, China; ^5^Department of Pharmacy, COMSATS University Islamabad, Lahore Campus, Lahore, Pakistan; ^6^The Second Department of General Surgery, Shaanxi Provincial People's Hospital, Xi'an, China

**Keywords:** colorectal cancer, ABL1, mutation, TGF-β1, PI3K/Akt

## Abstract

Colorectal cancer (CRC) is one of the most commonly diagnosed cancers worldwide. ABL1 (c-Abl) is a non-receptor tyrosine kinase, whose role, and molecular mechanism in CRC remain largely unclear. The aim of this study was to elucidate the role of ABL1 to obtain information on colon cancer gene mutation. We analyzed the tissue samples obtained from patients with CRC, CRC cell lines, and the immunodeficient mice. The proliferation, cell cycle, and apoptosis of CRC cells were examined. IPA software was used to analyze the molecules involved in CRC after ABL1 RNA interference. We found ABL1 was highly expressed in CRC tissues and cells. This high expression was associated with the TNM stage of CRC patients. In exon 8 of the ABL1 gene, we identified a novel mutation of C1222C deletion, which was related to the CRC stage. Depletion of ABL1 resulted in the inhibition of proliferation and escalation of apoptosis in two CRC cell lines, SW480, and HCT-116. Our *in vivo* study also demonstrated that depletion of ABL1 reduced CRC tumor progression. The results of the ingenuity pathway analysis indicated that the expression of 732 genes was upregulated and that of 691 genes was downregulated in mice transplanted with ABL1-downregulated CRC cells, among which we confirmed that depletion of ABL1 inhibited TGF-β1 via IRS1/PI3K/AKT pathway in CRC progression. These findings demonstrated that ABL1 plays an important role and that it can be a potential molecular target for CRC therapy.

## Introduction

Colorectal cancer (CRC) is a malignant tumor in the gastrointestinal tract, and it arises from the inner wall of the large intestine (the colon) (1). CRC is the third most common cancer worldwide, accounting for roughly 1.4 million new cases per year, and ~600,000 deaths per year, which makes it the fourth most common cause of cancer-related death globally and remains a huge challenge (2–4). In order to identify effective molecular targets for CRC diagnosis and potential interventions for CRC therapy, in-depth studies on the regulatory mechanism of CRC progression should be conducted.

The pathogenesis of CRC accompanies with genetic or epigenetic changes (5). Numerous genes and pathways, such as WNT, TGF-β, EGFR–RAS, ERK–MAPK, PI3K, and p53, have been demonstrated to be associated with CRC (6–10). ABL1, a proto-oncogene of c-Abl, encodes a non-receptor tyrosine kinase plays an important role in carcinogenesis, regulating cell adhesion, proliferation, differentiation, and apoptosis (11, 12). Studies have characterized ABL1 as an oncogene that promotes breast cancer cell proliferation and induces anchorage-independent growth under p53 deficiency in breast cancer cells (13–15). Craig et al. reported that inhibition of ABL1 by imatinib reduced the proliferation of lymphoma cells and prevented tumor formation in mice (16). However, the role and mechanism of ABL1 in CRC development and progression remain largely unclear.

The aim of this study was to elucidate the role of ABL1 using high-throughput DNA sequencing technology to obtain information on colon cancer gene mutation. We analyzed the variation in the expression of ABL1 among patients with CRC and in CRC cell lines. We additionally determined the effect of downregulating ABL1 on the proliferation, cell cycle progression, and apoptosis of CRC cells. Further, the effects of knockout of ABL1 in tumor and the molecular mechanisms of activated and suppressed downstream signaling pathways were assayed to elicit the mechanisms involved in CRC carcinogenesis.

## Materials and Methods

### Patients and Samples

Forty-eight patients with CRC were admitted at the Shaanxi People's Hospital (Shaanxi, China). Colorectal cancer was confirmed by histopathology or biopsy, based on which the clinical-pathological data of the subjects were evaluated. Formalin-fixed paraffin-embedded (FFPE) tissues were used as the study material. Tumor contents in the FFPE tissues were thoroughly checked at the Department of Histopathology, Shaanxi People's Hospital, and only FFPE tissue blocks with >30% tumor content were qualified. The study was approved by the Ethics Committee of Shaanxi People's Hospital. Written informed consent was obtained from all subjects participating in this study.

### DNA Extraction and Sequencing

Forty-eight specimens of colorectal cancer tissue with clinical liver metastases were collected. DNA was isolated using ALL prep DNA FFPE Kit (Qiagen, Germantown, MD). Genomic DNA was extracted by fully automated purification using Promega Maxwell (Promega, Madison, WI). The DNA concentration was measured fluorimetrically using the QuBit 2.0 DNA High Sensitivity Kit (Thermo Fisher Scientific, Waltham, MA). An ion torrent semiconductor chip sequencer was used to sequence common gene mutations in the tumors.

### Cell Culture and RNA Interference

CRC cell lines NCM460, Lovo, SW620, SW480, and HCT-116 were purchased from the Cell Bank of the Chinese Academy of Sciences (Shanghai, China). The cells were cultured in RP1640 medium supplemented with 10% heat-activated fetal bovine serum (Gibco, Gaithersburg, MD) and 1% penicillin-streptomycin (Gibco), at 37°C with 5% CO_2_.

ABL1, protein phosphatase 3 catalytic subunit alpha (PPP3CA), and TGF-β1 knockdown (KD) lentiviruses were generated using pFU-GW-GFP-RNAi vector by inserting shABL1, shPPP3CA, and shTGF-β1 sequence. Empty pFU-GW-GFP vector was used as vector control (shCtrl in CRC cells or NC mice). The RNAi sequence of ABL1, PPP3CA, and TGF-β1 were 5′-CGTTCTATATCATCACTGA-3′, 5′-ATATACGCGTTCTGAATACTT-3′, 5′-GATTATCGA CATGGAGCTG-3′, respectively. SW480 and HCT-116 cells were plated in a 24-well-plate and incubated at 37°C with 5% CO_2_ for 24 h. A multiplicity of infection of 100 was added to infect Sw480 and HCT-116 cells overnight. The infection medium was then replaced with normal complete growth medium. Cells without infection were used as corresponding controls.

### Proliferation and Colony Formation Assay

Proliferation rate was determined using Bromodeoxyuridine (BrdU) cell proliferation ELISA kit (Abcam, Boston, MA). The optical density of each sample was measured at 450 nm using a Synergy H1 microplate reader (Biotek, Winooski, VT).

For the clonogenic assay, SW480 and HCT-116 cells were plated onto 6-well-plate and incubated in culture medium for 7 days. The cells were then fixed with 4% PFA and stained with 0.5% crystal violet (Sigma-Aldrich, St. Louis, MO) for 1 h at room temperature. The total number of colonies was counted when each clone contained more than 50 cells (size 0.3–1.0 mm).

### Flow Cytometric Analysis

For cell cycle analysis, cells were fixed in 4% PFA for 30 min at 4°C and treated with propidium iodide (PI, 100 μg/mL, Sigma-Aldrich) at room temperature for 10 min in dark. A total of 10,000 cells were analyzed by flow cytometry using a BD FACSCalibur system (Becton–Dickinson, El Paso, TX). The distribution of cell cycle phases was estimated using ModFit LT in Mac V3.0 software. Apoptosis was further determined by Annexin V (FITC-conjugated, Thermo Fischer Scientific, Miami, OK) and PI staining. Cells were immediately counted by flow cytometry.

### *In vivo* Study

BALB/c nude mice (female, aged 4 weeks) were purchased from Shanghai Ling Chang Biological Technology Co., Ltd. (Shanghai, China). The mice were housed in SPF-level laboratories with free access to food and water and accommodated for 1 week prior to any experiments. The animal study was performed in accordance with IACUC guidelines. shABL1/HCT-116 (KD) or shCtrl/HCT-116 (NC) cells (3 × 10^7^/200 μL) were subcutaneously injected to the left flank of the mice. At day 21 post-transplantation, mice were sacrificed and tumors were excised and weighed. The tumor volume was calculated using digital calipers with the following formula (17):

Tumor volume = Volume length (width)^2^/2.

### Ingenuity Pathway Analysis

To elucidate the role and action mechanism of ABL1 in CRC, after ABL1 KD, high throughput real-time PCR array was performed by Shanghai Genechem Co., Ltd. (Shanghai, China) and the data were analyzed using ingenuity pathway analysis (IPA) software to elucidate the affected molecules and signal pathways.

### Immunohistochemistry

For CRC 180-Point Tissue Microarray (HCol-Ade180Sur-07), which contained CRC tissues from 89 patients and the corresponding adjacent tissues (Table S4), was purchased from Shanghai Outdo Biotech Co. Ltd. (Shanghai, China). Briefly, the tissue microarray block was constructed by embedding a single tissue core (1.5 mm in diameter) was taken from each region in formalin-fixed paraffin-embedded CRC or adjacent tissue block using a Tissue Microarrayer (Beecher Instruments, Silver Spring, MD, USA) and was set to a blank recipient block pre-drilled with 1.5 mm holes.

The tissue microarray blocks and paraffin-embedded tumor sections were cut into 7-μm sections for immunohistochemical (IHC) analysis. Slides were deparaffinized and rehydrated as previously described (18). Followed by antigen retrieval in citrate buffer (10 mM Citric Acid, 0.05% Tween 20, pH 6.0) for 30 min in 100°C water bath. After washing with PBS, slides were incubated with PBST with 1% bovine serum albumin (Sigma-Aldrich) for 1 h. Slides were then incubated overnight at 4°C with anti-ABL1 antibody (1:50, ab15130, Abcam, Cambridge, MA), and developed using Mouse and Rabbit Specific HRP/DAB Detection IHC kit (ab64264, Abcam) following the manufacturer's instructions.

The selection of cut-off value to dichotomize the expression levels of ABL1 was based on previously reported method [49]: Briefly, the high expression level of ABL1 was defined from two criteria: (1) DAB staining showed equal or darker color compared to positive control; (2) The population of ABL1-positive cells was higher than 70%. All cases were independently evaluated and diagnosed by two senior pathologists (Y. M and L. Y), who were blinded to the pathologic diagnosis. Cases with any disagreement were reviewed simultaneously by the original two pathologists and a senior pathologist (J. W) until they reach a consensus.

### Western Blot

The western blotting assay was performed by well-established protocols as previously described (19). Primary antibodies used in this study were anti-ABL1 antibody (1:500, ab85947, Abcam, Cambridge, MA), anti-Bcl-2 antibody (1:300, BCL/10C4, Biolegend, San Diego, CA), Anti-Bcl-xl antibody (1:500, sc-136207, Santa Cruz Biotechnology, Dallas, TX), anti-Bax antibody (1:300, 2D2, Biolegend), anti-β-actin antibody (1:500, 2F1-1, Biolegend), anti-GAPDH antibody (1:500, FF26A/F9, Biolegend), anti-p27 antibody (1:300, sc-56338, Santa Cruz Biotechnology), anti-cyclin-D1 antibody (1:500, sc-8396, Santa Cruz Biotechnology), anti-IRS1 antibody (1:500, ab52167, Abcam), anti-AKT2 antibody (1:500, ab175354, Abcam), anti-PPP3CA antibody (1:10000, ab52761, Abcam), anti-TGFβ1 antibody (1:100, ab92486, Abcam), anti-MAP2K2 antibody (1:500, sc-81473, Santa Cruz Biotechnology), anti-PI3K-p11a antibody (1:1000, ab151549, Abcam). Secondary antibodies used were: anti-mouse IgG HRP-conjugated secondary antibody (1:5000, sc-516102, Santa Cruz Biotechnology), and anti-rabbit IgG HRP-conjugated secondary antibody (1:5000, sc-2357, Santa Cruz Biotechnology).

### Reverse Transcription-Polymerase Chain Reaction

The mRNA level was measured using real-time polymerase chain reaction. Briefly, Total RNA was extracted from cultured cells using TRIzol Reagent (Thermo Fisher Scientific), and cDNA synthesis was performed using the QuantiTect Reverse Transcription Kit (Qiagen). The primers used were as follows: ABL1 sense: 5′-CATCACGCCAGTCAACAGTCT-3′ and antisense: 5′-ACACCCTCCCTTCGTATCTCAG-3′. GADPH sense: 5′-TGACTTCAACAGCGACACCCA-3′, antisense: 5′-CACCCTGTTGCTGTAGCCAAA-3′. The real-time PCR was carried out by using RT2 SYBR® Green qPCR Mastermixes (Qiagen) according to the manufacturer's instructions. All PCRs were performed in triplicate. ΔΔCt method was used to calculate the relative expression levels.

### Statistical Analysis

Statistical analyses were performed using SPSS 22 (SPSS Inc., Chicago, IL). χ^2^-test was used to investigate the possible relationships between ABL1 expression and clinic pathological characteristics. Mann–Whitney *U*-test was used to compare the difference in ABL1 protein expression between paired colon cancer and adjacent normal colon tissues. Survival analysis was performed using the Kaplan–Meier curve and the log-rank test. The values are expressed as mean ± SD. Comparisons between two groups were conducted using Student's *t*-test. All experiments were carried out in triplicate. Results with *p* < 0.05 were considered statistically significant.

## Results

### Highly Expressed ABL1 in CRC Tissue Is Associated With Poor Clinical Outcome

**Figure 1:**
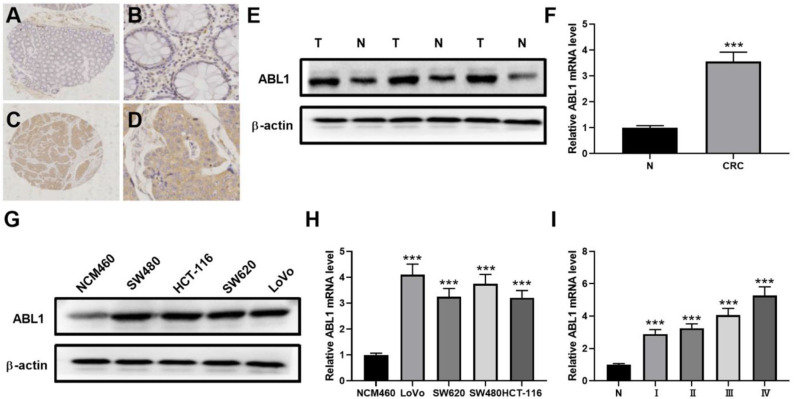
ABL1 is highly expressed colorectal cancer tissue and cell lines. **(A–D)** Representative IHC staining of ABL1 in normal **(A,B)** and CRC **(C,D)** tissues. **(A,C)**, 40 X; **(B,D)**, 200 X. **(E)** Western blot analysis of ABL1 expression in CRC tumor (T) and adjacent normal (N) tissues. **(F)** Real-time PCR analysis of ABL1 expression in CRC tumor (CRC) and adjacent normal (N) tissues. ***p < 0.001. **(G)** Western blot analysis of ABL1 expression in CRC cell lines. **(H)** Real-time PCR analysis of ABL1 expression in CRC cell lines, relative expression was normalized to NCM460 cells. ***p < 0.001. **(I)** Real-time PCR analysis of ABL1 expression in CRC tumors at different clinical stages, relative expression was normalized to adjacent normal (N) tissues. ***p < 0.001.

To verify the existence of ABL1 in CRC tissues, we compared CRC tissues and their adjacent non-cancerous tissues by IHC staining. Our results showed that the immunostaining of ABL1 was significantly higher in CRC tissues compared with adjacent normal colon tissues (*p* < 0.05, Figures 1A–D, Table 1). Our western blot and real-time PCR results confirmed the much higher expression level of ABL1 in CRC tissues compared to normal tissues (Figures 1E,F). Similarly, the expression of ABL1 was significantly increased in different CRC cell lines compared with that in the normal colon cell line NCM460 (Figures 1G,H). Remarkably, the expression of ABL1 was significantly (*P* < 0.001) increased in the advanced stages (stage II/III/IV) of CRC compared with the early stages (stage I) and non-cancerous tissues (Figure 1I). With a median follow-up of 31 months (ranging from 2 to 62 months), our survival analysis showed that the patients with high ABL1 expression (31 death) had a lower survival rate compared to patients with low ABL1 expression (16 death) (*p* = 0.034, Figure S5). These results suggested that ABL1 is a potential oncogene *ABL1* and that its expression was positively associated with the clinical stage in patients with CRC.

**Table 1:** Expression of ABL1 in colon cancer and adjacent tissues.

**Variables**	**No.**	**Expression levels**			***P***
		**Neg.**	**Low**	**High**	
Normal^*^	90	58	15	17	
Colon cancer	90	12	17	61	<0.001

* *Adjacent normal colon tissues, Neg., negative*.

### C1222C Deletion in Exon 8 of *ABL1* in Relation to the TNM Stage

**Figure 2:**
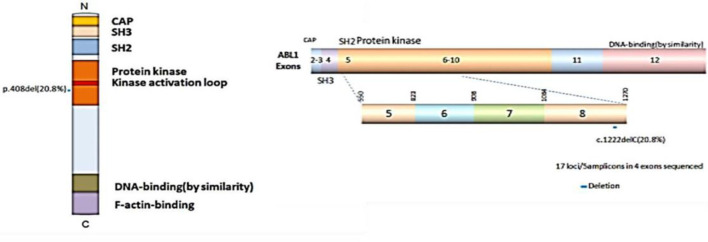
Structural domain and C1222C deletion in exon 8 of the ABL1 gene.

Previous studies have shown the mutations of the ABL1 gene are of major clinical relevance (20, 21). To study the possible mutations in patients with CRC, we performed DNA sequencing. Our results indicated that a mutation of ABL1 was present in 10 (3 males and 7 females) of 48 patients with CRC (31 females and 17 males). This mutation occurred in exon 8 of the ABL1 gene and all mutations were found to be deletion of the C1222C nucleotide sequence within this exon. The incidence rate of mutation was 22.6% in females and 17.6% males (Table 2, Figure 2). Additionally, the analysis of 10 patients with mutations and the corresponding stages revealed that 2 patients were in stages 1–2 and 8 in stages 3–4. The TNM stage was a significant risk factor for C1222C deletion. The results suggested that C1222C deletion is involved in CRC carcinogenesis.

**Table 2:** C1222C deletion in exon 8 of ABL1.

**Variables**	**Expression levels**			**OR(95% CI)**	***P***
	**No.**	**No mutation**	**Mutation**		
Gender				1.361(0.302–6.128)	0.687
Female	31	24	7		
Male	17	14	3		
TNM stage				17.714(3.069–102.062)	<0.001
1–2	33	31	2		
3–4	15	7	8		

### Interference of *ABL1* Decreased the Proliferation and Enhanced the Apoptosis of SW480 and HCT-116 Cells

**Figure 3:**
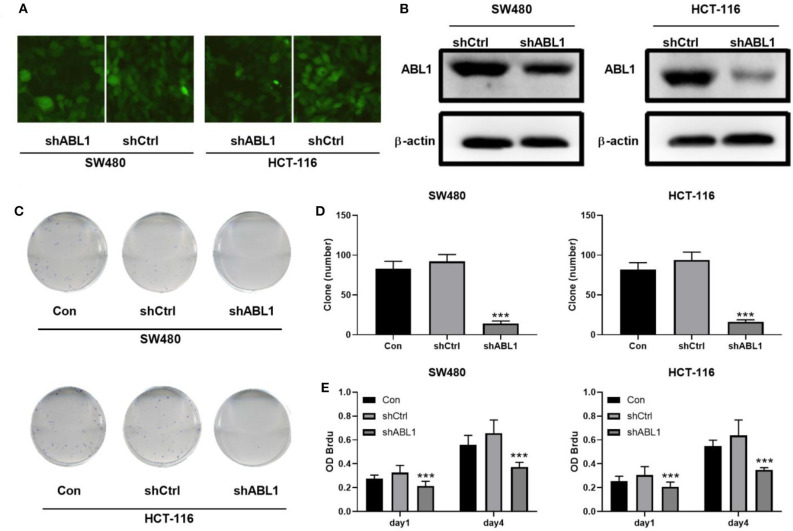
Depletion of ABL1 decreases the proliferation of CRC cells. **(A)** Representative fluorescent images showing GFP-positive cells after infection. Images were taken under 100 X. **(B)** Western blot detects the ABL1 expression in cells transfected infected with shCtrl or shABL1. **(C)** Representative images of colony formation assay using CRC cells infected with shCtrl or shABL1. Cells without infection was used as control (Con). **(D)** Quantification of colony numbers. Data are shown as mean ± SD. ***p < 0.001 compared with Control. **(E)** BrdU assay detects the proliferation of SW480 and HCT-116 cell lines infected with shCtrl or shABL1. Cells without infection was used as control (Con). ***p < 0.001 compared with Control.

**Figure 4:**
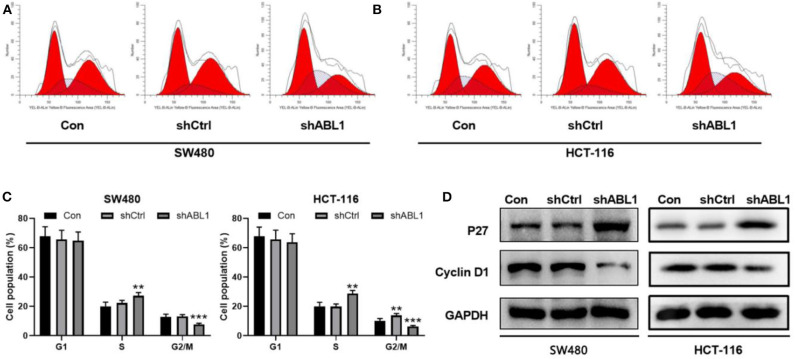
Depletion of ABL1 causes cell cycle arrest and apoptosis in SW480 and HCT-116 cells. **(A,B)** Representative cell cycle analysis of SW480 **(A)** and HCT-116 **(B)** infected with shCtrl or shABL1. Cells without infection was used as control (Con). **(C)** Quantification of cell cycle distribution in G1, S, G2/M phases. **p < 0.01; ***p < 0.001 compared to control group. **(D)** Western blot analysis of p27 and Cyclin D1 expressions in CRC cells infected with shCtrl or shABL1. Cells without infection was used as control (Con).

To further investigate the role of ABL1 in CRC carcinogenesis, we used lentivirus vector to downregulate ABL1 expression in SW480 and HCT-116 cells. After infection, both cell lines showed 70–80% of average GFP-positive rate (Figure 3A). Our western blot results showed a significant decrease of ABL1 protein level (Figure 3B), indicating a successful downregulation after RNA interference. To evaluate the proliferation of CRC cells after ABL1 depletion, we performed a clonogenic assay (Figure 3C). Compared with the control group, the number of clones in the shABL1 group was obviously decreased (Figure 3D). Our BrdU proliferation assay confirmed that the proliferation of shABL1 cells was obviously reduced compared with that of the control cells (Figure 3E). Additionally, our flow cytometry results showed more cells were arrested in S phase after ABL1 depletion, while fewer cells in G2/M phases were found (Figures 4A–C), indicating downregulation of ABL1 inhibited cell cycle progression of CRC cells. To validate these data, we detected P27, a negative regulator of cell cycle progression, and found its expression was significantly increased in cells infected with shABL1 vector (Figure 4D). On the contrary, cyclin D1 was decreased in ABL1-depleted CRC cells (Figure 4D).

**Figure 5:**
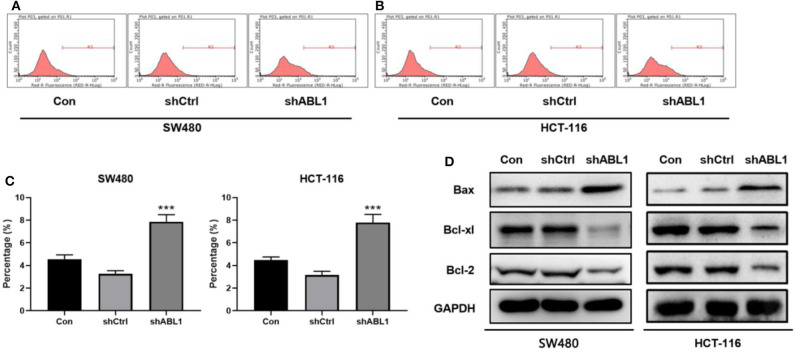
Depletion of ABL1 increases the apoptosis of CRC cells. Flow cytometry detected apoptosis of SW480 **(A)** and HCT-116 **(B)** cells infected with shCtrl or shABL1. Cells without infection was used as control (Con). **(C)** Quantification of apoptotic cells. Data are shown as mean ± SD. ***p < 0.001 compared with control. **(D)** Western blot analysis of Bax, Bcl-xl, and Bcl-2 expression in CRC cells infected with shCtrl or shABL1. Cells without infection was used as control (Con).

Next, we performed flow cytometric analysis to examine the apoptosis of ABL1-depleted CRC cells (Figures 5A,B). We found downregulation of ABL1 significantly increased apoptosis in CRC cells as compared with the control group (*p* < 0.05, Figure 5C). The expression of the apoptosis-related protein Bcl-2-associated X (Bax) was obviously increased while B-cell lymphoma-extra-large (Bcl-xl) and B-cell lymphoma 2 (Bcl-2) were remarkably decreased in the shABL1 group (Figure 5D). Taken together, these results suggest depletion of ABL1 increases the apoptosis of CRC cells.

### Depletion of ABL1 Inhibited CRC Tumor Growth *in vivo*

**Figure 6:**
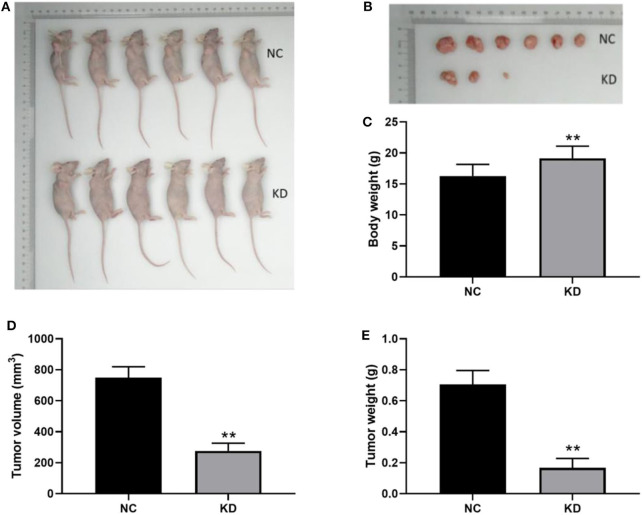
Depletion of ABL1 inhibits CRC tumor growth *in vivo*. **(A)** Representative image of mice injected with HCT-116 cells infected with shCtrl (NC) or shABL1 (KD). **(B)** Representative tumor images showing depletion of ABL1 decreased the size of CRC tumors. Body weight **(C)**, tumor volume **(D)**, and tumor weight **(E)** were measured at day 21 after inoculation. **p < 0.01 compared to NC group.

In order to examine the involvement of ABL1 in regulating CRC tumor growth, we inoculated HCT-116 cells infected with shCtrl (NC group) or shABL1 (KD group) into BALB/c nude mice (Figure 6A). As shown in Figure 6B, the tumor growth was remarkably inhibited in the KD group compared with the NC group. Interestingly, we also observed a significant bodyweight increase in the KD group (Figure 6C). As compared to control, ABL1 depletion caused a significant reduction in tumor volume (Figure 6D). The average tumor weight in KD xenografts was obviously lower than that in the NC group (233.68 ± 64.58 mg vs. 742.5 ± 89.41 mg, *p* < 0.01) (Figure 6E). Taken together, our animal experiments demonstrated that ABL1 knockdown could inhibit CRC tumor growth *in vivo*.

### ABL1 Interference Inhibited TGF-β1 via the PI3K/Akt/IRS1 Pathway and PPP3CA

To elucidate the molecular pathways regulated by ABL1 in CRC, we performed high throughput PCR array from xenografts in ABL1 KD or NC mice. Our IPA results identified 732 upregulated genes and 691 downregulated genes in xenografts from KD mice compared with those from NC mice. Further analysis revealed that these differentially expressed genes were involved in multiple biological functions and pathogenesis of multiple diseases (Figure S1).

**Figure 7:**
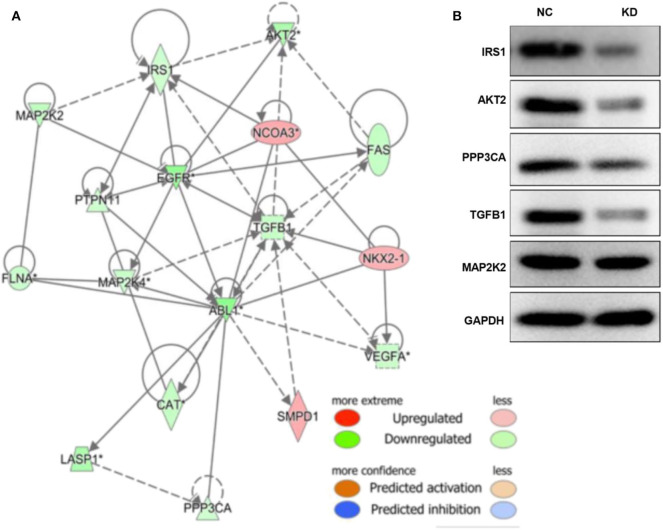
ABL1 interacts with PI3K/Akt and TGF-β1 pathways. **(A)** Molecule network generated by IPA showing interactions among ABL1, PI3K/Akt, and TGF-β1 pathways. Up- and down-regulated genes are shown in red and green, respectively. **(B)** Western blot analysis of predicted interactive proteins in xenografts from ABL1 knockdown (KD) or control (NC) mice. The gene of “*” is detected by multiple probes, and it is statistically significant.

As shown in Figures S2, S3, and Tables S1–S3, the TGF-β and PI3K/Akt pathways were inhibited by depletion of ABL1. The associated molecules of the two pathways, including TLR4, AKT2, IL4R, CAMK2D, PPP3CB, MAP2K2, PDIA3, IRS1, ITPR3, ABL1, ATF4, PPP3CA, CA2, CPT1A, CSRP1, CTSV, FN1, LAMP2, PTGS2, RUNX2, S100A4, and SPP1, were mapped with ABL1 gene to show a predicted interaction network (Figure 7A). To verify this interaction, we examined the levels of proteins in TGF-β and PI3K/Akt pathways in xenografts of NC and KD mice (Figure 7B). Our western blot results showed that knockdown of ABL1 significantly decreased IRS1, AKT2, PPP3CA, and TGF-β1 expression, while did not change the expression of MAP2K2 compared with those in the NC group.

**Figure 8:**
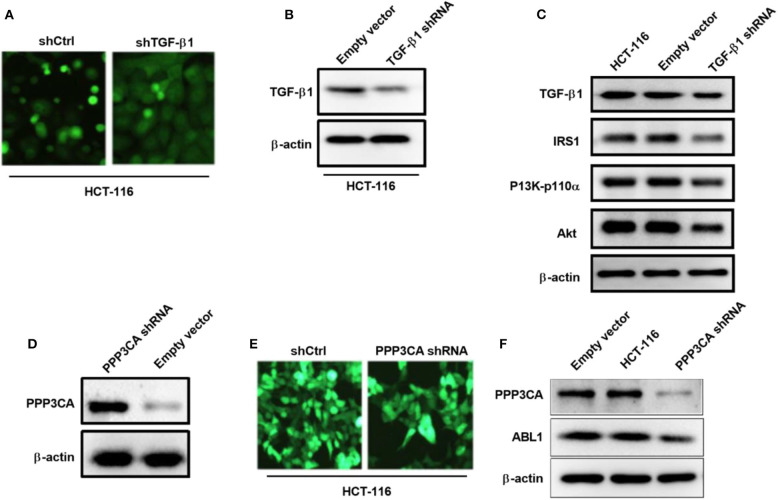
TGF-β1 knockdown deactivates PI3K/AKT pathway in HCT-116 cells. **(A)** Representative images of HCT-116 cells infected with GFP-containing lentivirus expressing shRNA against TGF-β1. GFP positive lentivirus with scramble shRNA was used as control. Images were captured under 200x magnification. **(B)** Knockdown of TGF-β1 was confirm by western blot. Proteins were extracted from HCT-116 cells at 24 h post-infection. β-actin was used as loading control. **(C)** Western blot examined IRS1/PI3K/Akt pathway in HCT-116 cells after knockdown of TGF-β1. **(D)** Western blot examined IRS1/PI3K/Akt pathway in HCT-116 cells infected with GFP-containing lentivirus expressing shRNA against PPP3CA. GFP positive lentivirus with scramble shRNA was used as control. **(E)** Representative fluorescent images of HCT-116 cells at 24 h post-infection. Images were captured under 200x magnification. **(F)** Expression of ABL1 after PPP3CA knockdown was determined by western blot. Proteins were extracted from HCT-116 cells at 24 h post-infection. β-actin was used as loading control.

To further verify the involvement of TGF-β1 in the regulation of PI3K/AKT pathway, we generated a TGF-β1-depletion HCT-116 cell line by lentivirus infection (Figure 8A), our western blot result showed that the expression of TGF-β1 was significantly downregulated after infection (Figure 8B). As expected, the key proteins in PI3K/AKT pathways were deactivated upon TGF-β1-depletion (Figure 8C), including IRS1, phospho-PI3K, and Akt. According to the findings in the previous study, the downregulated gene PPP3CA found in ABL1 KD mouse is involved in the regulation of the PI3K/AKT pathway (22). We next investigated the interaction between PPP3CA and ABL1 by establishing a PPP3CA knockdown cell line (Figures 8D,E). We found the depletion of PPP3CA significantly decreased the expression of ABL1 (Figure 8F). To validate the regulatory role of PPP3CA, we also examined the ABL1 expression in PPP3CA overexpressed cells, and found the expression of ABL1 was elevated by upregulated PPP3CA (Figure S4), indicating PPP3CA is a positive regulator of ABL1.

## Discussion

As a ubiquitously expressed non-receptor tyrosine kinase, ABL1 has been reported to be associated with glioblastoma and breast cancer (12, 14). In this study, we examined the role of ABL1 in CRC progression. The results indicated that ABL1 might play an important role in CRC, which is associated with the mutation and expression of the *ABL1* gene.

We found the expression of ABL1 was remarkably elevated in CRC tissues and cell lines (Figure 1), which is corresponded to the survival rate among patients with CRC (Figure S5), indicating ABL1 is a potential oncogene in CRC (13–15). Moreover, the mutation of ABL1 was also elevated in CRC patients. Based on the results from previous studies, the mutation rate of the ABL1 gene is relatively higher in men than in women patients with CRC worldwide (3, 23). In a previous study, the *ABL1* gene was found to be mutated in 0.9% of patients with CRC at Sir Ganga Ram Hospital, Delhi, India (5). In the present study, the mutation rate was much higher (10/48, 20.83%) in CRC patients accepted in our hospital. The different races of patients from different regions of the world reported in the two studies could be a possible reason causes the difference of ABL1 mutation rates (2–4). Gene mutations are often involved in tumorigenesis, the clustered deletions were found in *ABL1, NOTCH1, RET, STK11, GNA11*, and *JAK3* genes in CRC, melanoma, and non-small cell lung cancers (24). Additionally, the ABL1 mutation data in TCGA showed that an average mutation rate of ABL1 is 7.17% (32/448) in COAD patients. The high mutation rate is consistent with the findings in this study that ABL1 mutation correlates with the oncogenesis of CRC. To the best of our knowledge, the present study presents a novel mutation in exon 8, in which C1222C deletion occurred. This deletion was relatively higher in female patients than in male patients. The higher distribution of this deletion at the higher TNM stage in patients with CRC suggests that this deletion might be related to tumorigenesis of CRC. However, further investigation with larger sample size is needed to elucidate the relationship and mechanism between C1222C deletion and CRC progression.

To determine the role of ABL1 in CRC progression, we downregualted ABL1 expression in CRC cell lines and found that the cell cycle was arrested at S phase (Figure 4). These observations are consistent with previous reports that the number of cells in S phase was increased when ABL1 was inhibited by imatinib or STI571 in U2OS, HeLa, and A549 cells (25, 26). It is well-reported that p27 inhibits G1/S transition of the cell cycle, while cyclin D1 is a key regulator of cell entry into the S phase, allowing cells to enter the S phase smoothly from the G1 phase (27, 28). Our study provides direct evidence that ABL1 interference increased p27 expression and decreased in cyclin D1 expression (Figure 4), which is similar to the increased p27 expression and decreased cyclin D1 expression found in cells treated with nilotinib, an ABL1-specific inhibitor (29, 30). However, previous studies showed that when ABL1 expression was inhibited by nilotinib, the number of cells in the G0/G1 phase was increased while the number of cells in S and G2/M phases was decreased (30), which is contradictory to the finding in this study that downregulation of ABL1 arrested CRC cells at S phase. This might be due to the varied function of ABL1 in different tissues and cell types (25).

ABL1 controls cell apoptosis via downstream molecules such as PUMA, Bax, and p73, as well as by changing membrane potential (31, 32). Depletion of ABL1 induced apoptosis of CRC cells observed in this study is consistent with the findings of these studies. Studies have reported that ABL inhibitor danusertib treatment significantly decreased the expression of Bcl-xl and Bcl-2 while increasing the expression of Bax (33, 34). Similarly, we found increased Bax expression and decreased levels of Bcl-2 and Bcl-xl after downregulation of ABL1 in CRC cells (Figure 5), indicating ABL1 is involved in the regulation of apoptosis in CRC cells.

Furthermore, information obtained using the IPA indicated that after ABL1 knockdown, the expressions of numerous genes involved in cell proliferation, migration, invasion, differentiation, death, and survival were affected (Figures S1–S3, Tables S1–S3). The results demonstrated that ABL1 might play a pivotal role in CRC progression (11, 12). Especially, the TGF-β and PI3K/Akt pathways were inhibited after ABL1 interference.

It has been reported that ABL1 regulates TGF-β signaling (35), which is associated with tumor progression by modulating angiogenesis in CRC, resulting in poor prognostic outcome (36–38). Studies have demonstrated that treatment with an ABL1 inhibitor significantly reduced the TGF-β level (39, 40). Similarly, we found the expression of TGF-β1 was significantly inhibited after ABL1-depletion (Figure 7). This indicated that ABL1 is a positive regulator of TGF-β signal pathways. As one of the TGF-β-affected downstream signals, the PI3K/AKT pathway plays a crucial role in tumorigenesis. It regulates the expression of proteins associated with proliferation, apoptosis, invasion, and metastasis of cancer cells (41, 42). Insulin receptor substrates (IRS), including IRS1 and IRS1, are a downstream messenger of the PI3K pathway (43). Our study provides novel evidence that ABL1 might interact with TGF-β1 via PI3K/Akt/IRS1 that is involved in CRC progression.

Calcineurin, a Ca^2+^- and calmodulin-dependent serine/threonine protein phosphatase, has been reported to promote intestinal tumor development and CRC tumorigenesis (22). The expression of calcineurin A specifically increases in human CRC cell lines (44). In the present study, we found that PPP3CA, which is also known as an alpha isoform of the calcineurin catalytic subunit (45), was inhibited after knockdown of ABL1 (Figure 7B). This finding provides novel evidence that ABL1 might interact with the *PPP3CA* oncogene in CRC carcinogenesis.

## Conclusion

In conclusion, we found a high level of ABL1 expression in CRC tissue and cells, which was associated with the TNM stages. A novel mutation of C1222C deletion in exon 8 of the *ABL1* gene was found and was associated with the CRC stage. Depletion of ABL1 decreased the growth of CRC cell lines both *in vitro* and *in vivo* by inhibiting TGF-β pathway. These results demonstrated novel understandings of the function of ABL1 during the progression of CRC, thus provides a clinically viable strategy for CRC therapy.

## Data Availability Statement

The raw data supporting the conclusions of this article will be made available by the authors, without undue reservation.

## Ethics Statement

The studies involving human participants were reviewed and approved by Ethics Committee of Shaanxi People's Hospital. The patients/participants provided their written informed consent to participate in this study. The animal study was reviewed and approved by Ethics Committee of Shaanxi People's Hospital.

## Author Contributions

All authors have a significant scientific contribution to all aspects of this study.

## Conflict of Interest

The authors declare that the research was conducted in the absence of any commercial or financial relationships that could be construed as a potential conflict of interest.
