# Supplementary material for: C1222C Deletion in Exon 8 of ABL1 Is Involved in Carcinogenesis and Cell Cycle Control of Colorectal Cancer Through IRS1/PI3K/Akt Pathway
Source: Front Oncol. 2020 Aug 11;10:1385. doi: 10.3389/fonc.2020.01385 (PMC7433659; doi:10.3389/fonc.2020.01385)
Supplement: Supplementary file 1 [file Data_Sheet_1.docx]

**Supplementary data**

**
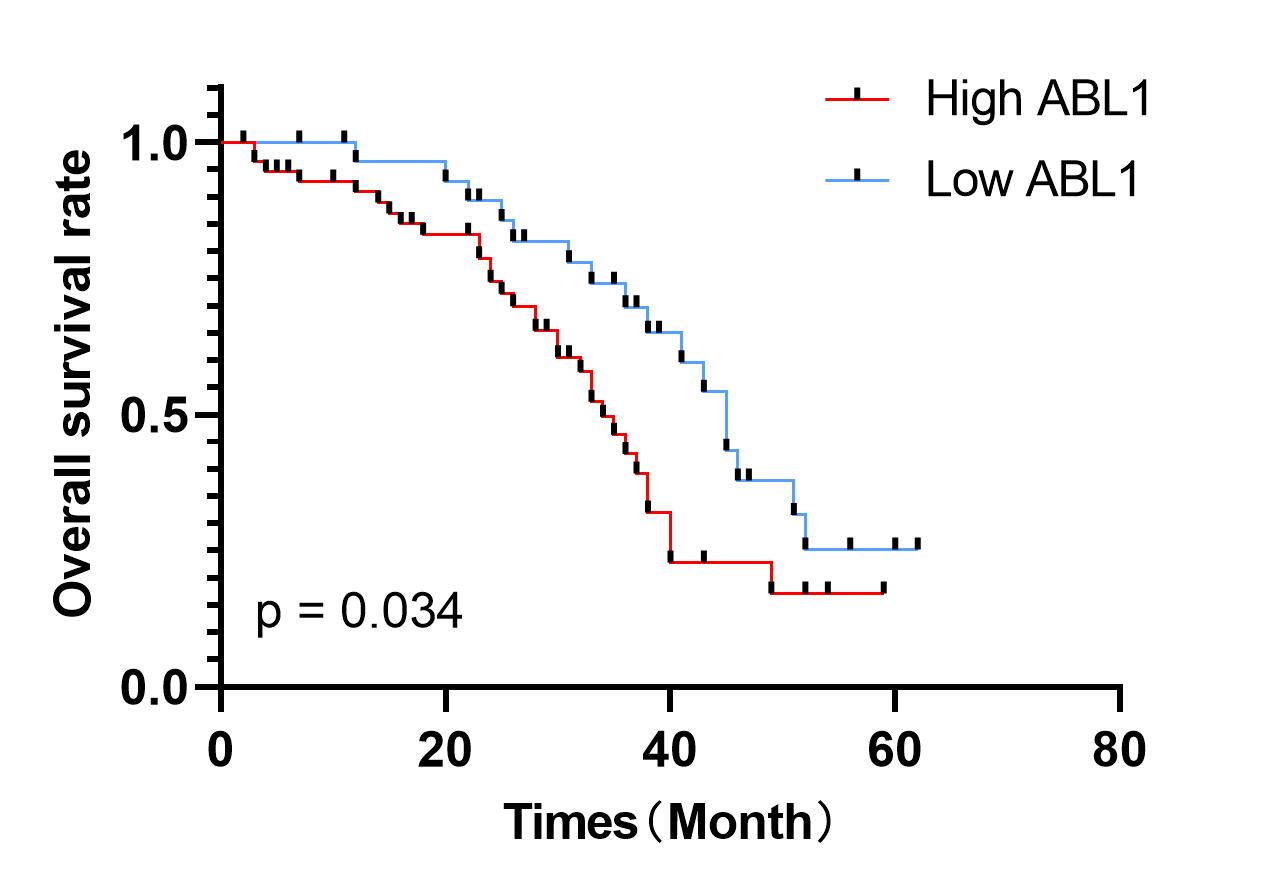
**

**Figure S1.** **Kaplan-Meier curve of patients with high or low ABL1 expression.**


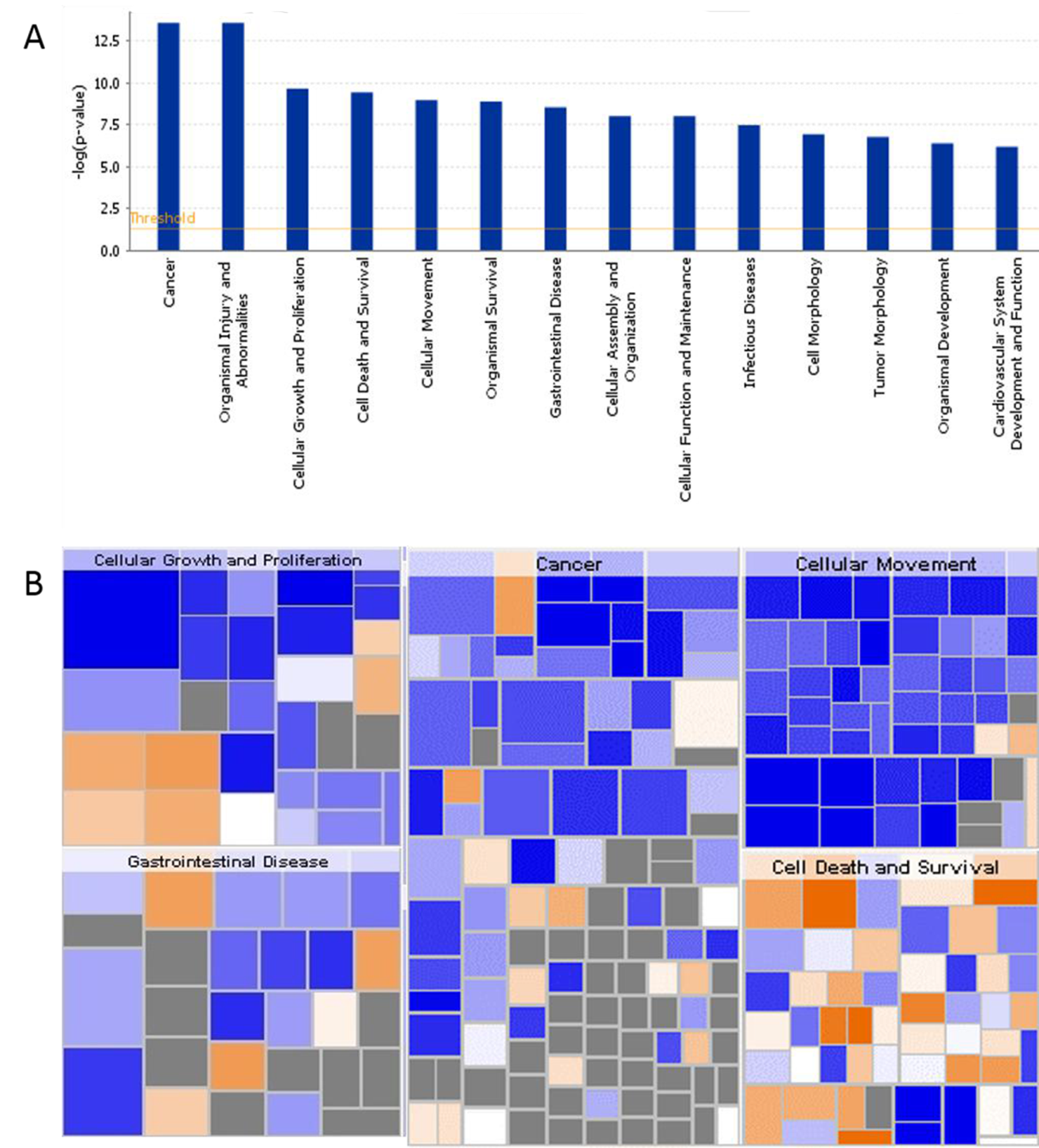


**Figure S2. Enrichment analysis of genes activated or inhibited by ABL1-depletion.** (A) histogram. (B) hotspot. Orange represents Z-score > 0, blue represents Z-score < 0, gray represents no Z-score value. Z-score > 2 represents that the function is significantly activated and Z-score < -2 represents that the function is significantly inhibited.


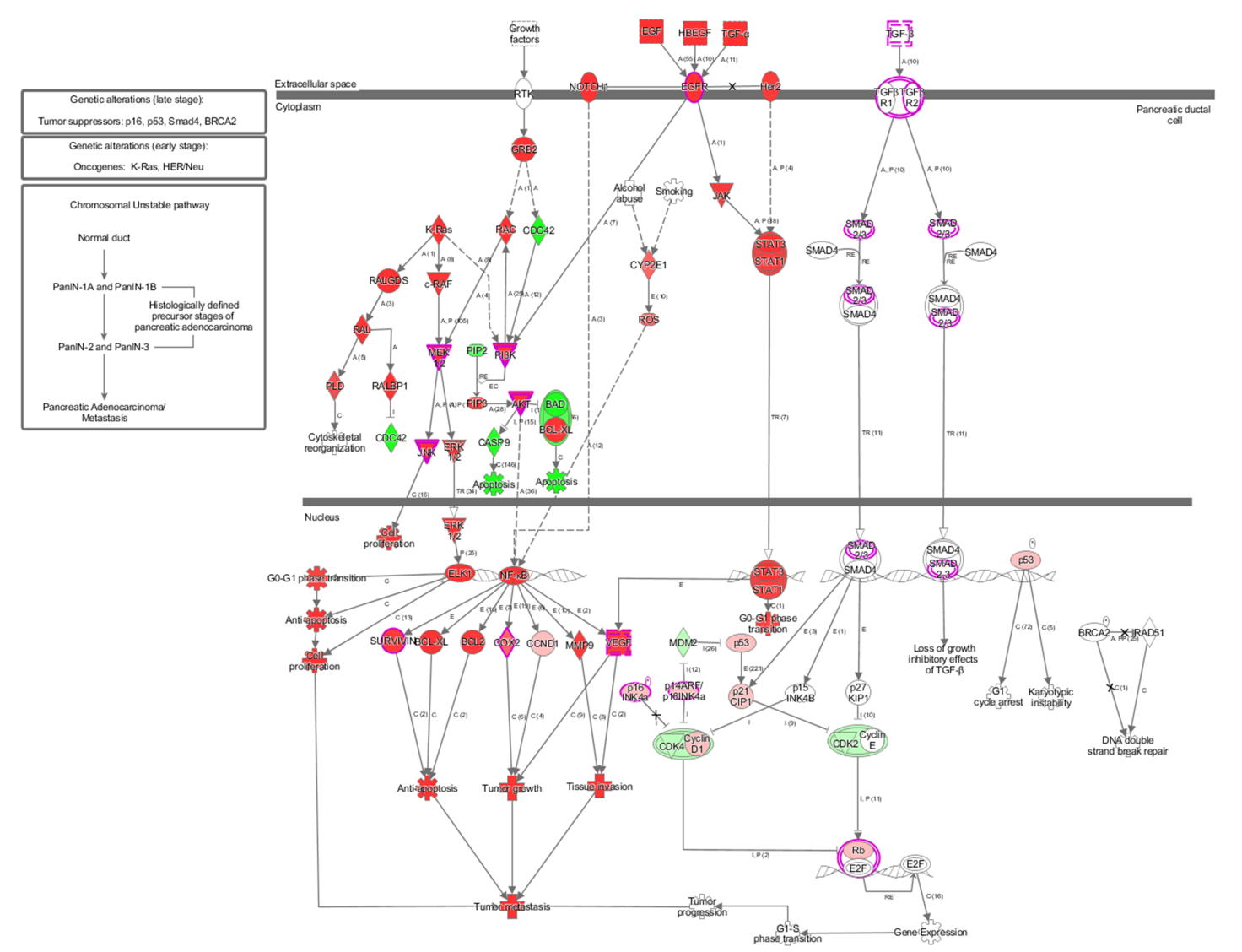


**Figure S3. Activated or inhibited signaling pathways after ABL1 interference.**


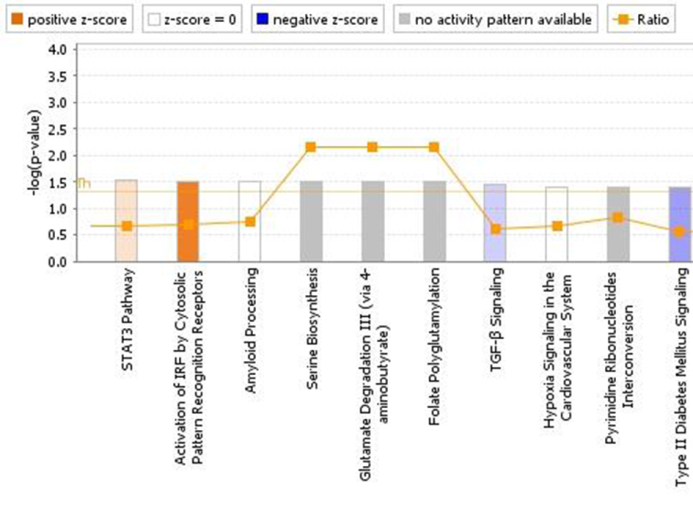


**Figure S4. Inhibition of the TGF-β signaling after ABL1-depletion.**

**
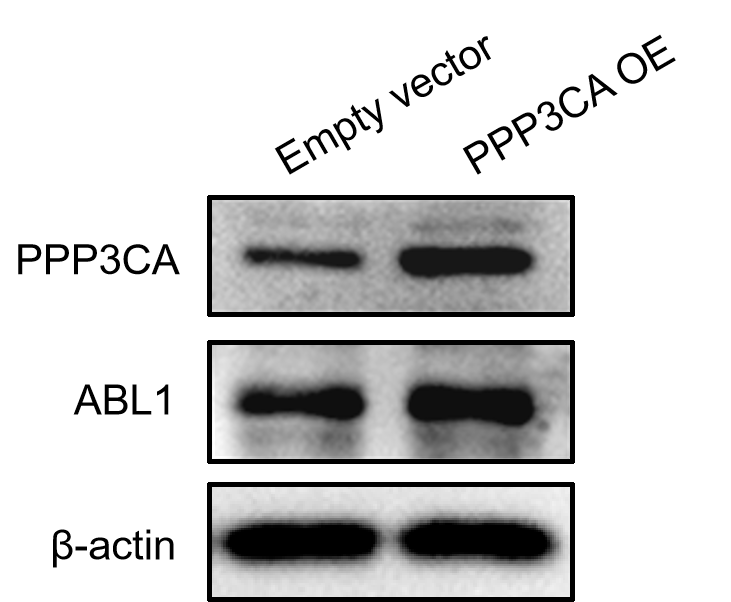
**

**Figure S5. Western blot detection of ABL1 expression in PPP3CA overexpressed HCT-116 cells.**

**Table S1.** **TGF-β signaling inhibited by ABL1-depletion**

| Ingenuity Canonical Pathways | -log  (p-value) | Ratio | z-score | Molecules | Counts of Antibody List |
| --- | --- | --- | --- | --- | --- |
| Ephrin B Signaling | 1.52 | 0.123 | -0.447 | CFL2,CXCR4,EFNB1,GNG2,  GNA11,ITSN2,EPHB3,GNG12,  LIMK1 | 0 |
| STAT3 Pathway | 1.52 | 0.123 | 0.333 | MAP2K4,TGFBR2,MAP2K2,  TGFBR3,SOCS6,MAPK9,  TNFRSF11A,CDC25A,EGFR | 4 |
| Activation of IRF by Cytosolic Pattern Recognition Receptors | 1.51 | 0.129 | 1.414 | MAP2K4,IFIH1,DDX58,MAPK9,  IKBKAP,IRF9,IFIT2,IFNAR1 | 2 |
| Amyloid Processing | 1.5 | 0.137 | NA | PRKACB,CAPN8,AKT2,CAPN1,  PSEN2,CAPN2,PRKAR1A | 1 |
| Serine Biosynthesis | 1.5 | 0.4 | NA | PSAT1,PHGDH | 0 |
| Glutamate Degradation III (via 4-aminobutyrate) | 1.5 | 0.4 | NA | SUCLG2,GAD1 | 0 |
| Folate Polyglutamylation | 1.5 | 0.4 | NA | MTHFD1L,SHMT2 | 0 |
| TGF-β Signaling | 1.45 | 0.115 | -0.632 | MAP2K4,TGFBR2,SMAD2,  MAP2K2,TGFB1,RUNX2,  MAPK9,SMURF2,PITX2,ACVR1B | 5 |
| Hypoxia Signaling in the Cardiovascular System | 1.4 | 0.123 | NA | VEGFA,UBE2D2,UBE2J1,UBE2A,  UBE2B,UBE2N,UBE2V2,ATF4 | 1 |
| Pyrimidine Ribonucleotides Interconversion | 1.4 | 0.156 | NA | NME4,NME1,AK9,NME7,  ENTPD7 | 0 |

**Table S2 Genes involved in TGF-β1 pathway**

| UR | EFC | MT | PAS | Activation z-score | p-value  of overlap | TM in dataset | Mechanistic Network | CAL |
| --- | --- | --- | --- | --- | --- | --- | --- | --- |
| ESR1 |  | ligand-dependent nuclear receptor |  | -0.263 | 2.95E-27 | **↓**ABLIM1… all 172 | 475 (18) | 26 |
| HNF4A |  | transcription regulator |  | -1.484 | 6.96E-16 | **↑**AAMDC,  … all 200 | 307 (9) | 12 |
| TGFB1 | -2.466 | growth factor | Inhibited | -2.667 | 1.57E-14 | **↓**ABCA1… all 167 | 425 (20) | 24 |
| TP53 |  | transcription regulator |  | 0.818 | 1.58E-13 | **↓**ACSL3,  …all 146 | 479 (21) | 26 |
| ATF4 | -2.003 | transcription regulator | Inhibited | -4.176 | 6E-13 | **↓**ASNS…  all 33 | 302 (10) | 7 |
| ERBB2 |  | Kinase |  | -1.146 | 2.36E-12 | **↓**ABHD5,  … all 82 | 345 (19) | 18 |
| UCP1 |  | transporter | Inhibited | -3.421 | 2.11E-11 | **↓**ASNS, …  all 31 |  | 3 |
| MYC |  | transcription regulator |  | -1.337 | 8.49E-11 | **↑**ADARB1,  … all 109 | 467 (21) | 24 |
| IFNG |  | Cytokine |  | 1.323 | 2.08E-09 | **↓**ABCA1,… all 123 | 362 (18) | 19 |
| KRAS |  | Enzyme | Inhibited | -2.294 | 8.09E-09 | ABCA1, …  all 49 | 337 (17) | 15 |
| beta-estradiol |  | chemical - endogenous mammalian | Inhibited | -2.446 | 1.23E-08 | **↓**ABCA1,  … all 155 | 510 (18) | 24 |
| ALDH2 |  | Enzyme | Activated | 3.293 | 1.25E-08 | **↓**ATF4,… all 11 |  | 0 |
| IFNL1 |  | cytokine | Activated | 3.444 | 1.76E-08 | **↑**APOL6,  … all 19 | 336 (15) | 2 |
| EIF2AK3 |  | Kinase | Inhibited | -2.423 | 2.40E-08 | **↓**ASNS,  … all 22 | 327 (20) | 5 |
| TNF |  | Cytokine |  | -0.677 | 2.65E-08 | **↓**ABCA1,  … all 146 | 454 (20) | 25 |
| tosedostat |  | chemical drug | Inhibited | -3.434 | 3.24E-08 | **↓**ASNS,  … all 12 |  | 3 |

**Table S3. PI3K/AKT signaling inhibited by ABL1-depletion**

| Ingenuity Canonical Pathways | -log  (p-value) | Ratio | z-score | Molecules | Counts  of Antibody List |
| --- | --- | --- | --- | --- | --- |
| Glycine Betaine Degradation | 0.336 | 0.1 | NA | SHMT2 | 0 |
| Chondroitin Sulfate Biosynthesis | 0.335 | 0.069 | NA | GXYLT1,HS2ST1,HS3ST1,  NDST1 | 0 |
| RhoGDI Signaling | 0.332 | 0.0636 | 0.707 | ARHGAP5,RHOG,CFL2,  ACTA2,GNG2,GNA11,  PIKFYVE,RDX,ARHGAP1,  GNG12,LIMK1 | 0 |
| Lipid Antigen Presentation by CD1 | 0.33 | 0.0769 | NA | AP2M1,PDIA3 | 1 |
| PI3K/AKT Signaling | 0.33 | 0.0645 | -2.236 | TSC1,RHEB,AKT2,MAP2K2,  FOXO1,GDF15,PTGS2,EIF4EBP1 | 5 |
| Antiproliferative Role of Somatostatin Receptor 2 | 0.327 | 0.0667 | -1 | MAP2K2,PTPN11,IRS1,  GNG2,GNG12 | 2 |
| Reelin Signaling in Neurons | 0.321 | 0.0652 | NA | MAP2K4,PTPN11,IRS1,  ITGA6,MAPK9,ITGA1 | 3 |
| Factors Promoting Cardiogenesis in Vertebrates | 0.321 | 0.0652 | NA | TGFBR2,SMAD2,CCNE2,  TGFB1,TGFBR3,ACVR1B | 4 |
| Death Receptor Signaling | 0.321 | 0.0652 | 0 | MAP2K4,ACTA2,TNFSF10,  PARP11,FAS,LIMK1 | 2 |
| Role of PI3K/AKT Signaling in the Pathogenesis of Influenza | 0.317 | 0.0658 | -1.342 | AKT2,MAP2K2,PTPN11,  IRS1,IFNAR1 | 3 |
| Neurotrophin/TRK Signaling | 0.317 | 0.0658 | -2.236 | MAP2K4,MAP2K2,PTPN11,  IRS1,ATF4 | 2 |
| Dermatan Sulfate Biosynthesis | 0.311 | 0.0667 | NA | GXYLT1,HS2ST1,HS3ST1,NDST1 | 0 |
| Production of Nitric Oxide and Reactive Oxygen Species in Macrophages | 0.309 | 0.0622 | -0.577 | APOL1,MAP2K4,TLR4,AKT2,  RHOG,PTPN11,IRS1,CAT,  MAP3K1,MAPK9,SERPINA1,  TNFRSF11B | 4 |

**Table S4. Correlation between the ABL1 expression and clinicopathological features**

| Variable | ABL1 Expression | |
| --- | --- | --- |
|  | Low | High |
| Age |  |  |
| <55 | 5 | 21 |
| ≥55 | 20 | 43 |
| Gender |  |  |
| Male | 16 | 30 |
| Female | 15 | 28 |
| TNM stage |  |  |
| Ⅰ-Ⅱ | 26 | 5 |
| Ⅲ-Ⅳ | 19 | 39 |
